# The Efficiency Assessment of Dental Units Using Data Envelopment Analysis Approach: The Case of Iran

**Published:** 2017-04

**Authors:** Mohsen BAROUNI, Mohammad Reza AMIRESMAIELI, Arash SHAHRAVAN, Saeed AMINI

**Affiliations:** 1. Health Services Management Research Center, Institute for Futures Studies in Health, Kerman University of Medical Sciences, Kerman, Iran; 2. Health Management, Policy Making and Economics Department, Kerman University of Medical Sciences, Kerman, Iran; 3. Endodontology Research Center, Kerman University of Medical Sciences, Kerman, Iran

**Keywords:** Efficiency, Data envelopment analysis (DEA), Decay, Missing, Filled teeth (DMFT)

## Abstract

**Background:**

During the last decades, the number of dentistry units increased significantly across the country. The aim of this study was to assess the efficiency of dental units of Iran provinces regarding dental health inputs and outputs using Data Envelopment Analysis approach.

**Methods:**

In this applied descriptive-analytical study, the study population included all of Iran 31 provinces. The output variables included DMFT and DMFT indices of 6–12 yr old students. The data about DMFT and DMFT indices were taken from 2013 Nationwide School Pupils Screening Program. Input variables included active dental chairs located in the public sector, general dentists of public sector, general and specialist dentists of private sector by different provinces. The data were analyzed using Deap software version 2.1.

**Results:**

The lowest amount of scale efficiency was for Tehran Province (0.204) followed by Isfahan Province (0.205). Provinces of Isfahan, Razavi Khorasan, Kerman, Zanjan, Hamedan, Kordestan, Golestan, Yazd and Tehran, Iran had decreasing return to scale and provinces of Gilan, West Azerbaijan, Mazandaran, Fars, Kermanshah, Markazi, Lorestan, Qazvin, Sistan-and-Baluchestan, Bushehr, Alborz, Hormozgan and Khuzestan had increasing return to scale.

**Conclusion:**

Despite provinces of Isfahan, Razavi Khorasan, Kerman, Zanjan, Hamedan, Kordestan, Golestan, Yazd and Tehran which had a better situation in terms of the number of dentistry chairs, public dentists, general and specialist dentists of private sector than other provinces, they had decreasing return to scale. Investment in dental primary health care, preventive and educational programs can be more cost-effective.

## Introduction

One of the main topics in Iran development programs is health and wellbeing of Iranian population. For example, Iran constitution has identified health care services as an essential need and has obliged governments to mobilize all of their resources, facilities and capacities to provide, maintain and promote health of people (1). Regarding the high impact of investment in health care on workforce productivity, it is necessary to allocate adequate resources and use them efficiently (2).

On the other hand, different studies in developing countries including Iran indicate that more than half of health resources are wasted and limited resources are used inefficiently. In addition, public budgets are spent on services that do not have adequate appropriateness and effectiveness (3).

Efficiency assessment is the first step in performance assessment of different sectors of health system. Thus, measuring and assessing efficiency can provide logical framework for distribution of human and financial resources between different sectors (4). The combination of technical and allocative efficiency forms total efficiency. Technical efficiency means to use the lowest amount of inputs to produce a determined level of outputs or to produce more outputs using a fixed level of inputs. Allocative efficiency means employing inputs in the correct proportion in terms of their prices to produce a specified amount of outputs (5).

Technical efficiency is composed of two components: scale efficiency and managerial efficiency. In other words, technical efficiency is the result of multiplying scale efficiency in managerial efficiency. Scale efficiency is the ability of a unit to perform in or near most profitable scale to prevent the losses to resources. Managerial efficiency means hard working, effort and making good policy, employing proper staff and deploying the correct combination of production factors (6).

There are different methods to assess productivity and efficiency of corporations. These can be classified into two general groups of parametric (Stochastic Frontier Analysis or SFA) and nonparametric (Data Envelopment Analysis or DEA) methods. Parametric methods are based on econometrics models and microeconomics theories. Through panel data, production function is estimated by attention to the considered assumptions and then the efficiency of units is measured. However, DEA method is based on optimization using linear programming. The efficient frontier curve develops from a series of points that is determined by linear programming. The advantage of this method is in its freedom from explanation the type of production function. In addition, production factors and products can have different measurement units. DEA method determines a target unit for each inefficient observation (7). DEA is applied to assess the relative efficiency of decision-making units (DMUs) that have same duties, like assessment and comparison of organizational units of a ministry, schools, hospitals, bank branches and so on. In addition, DEA is applied for benchmarking, continuous improvement and strategic analysis (8).

In DEA method, there are virtual units named peer firms or reference collections that compare decision-making units (here dental health sector of provinces) with themselves to identify the efficiency rate of these decision-making units. These peer firms or reference collections have more outputs and lower inputs than decision-making units do.

DEA models in terms of recovery path are divided into categories of input-oriented and output-oriented. Input-oriented models emphasize on decrease in inputs and output-oriented models emphasize on increase in outputs to be efficient (9).

Because of its numerous advantages, DEA has attracted researchers’ attention. It can manage many inputs and outputs can compare decision-making units directly, its inputs and outputs can have different measurement units and finally does not need a hypothesis to relate inputs to outputs (10).

Numerous studies have been performed about efficiency estimation of different sectors of health care. For example, in a study at Yazd Province, relative efficiency of human resources of health centers was assessed using DEA method and the centers with low efficiency were identified and reported to policymakers to take improvement actions (11).

Another study in UK in 2000 assessed the efficiency of social dentistry services using DEA. Working hours of dental health practitioners, therapists, hygienists, and others were considered as inputs, and screening, prevention and treatment were considered as outputs. Relative efficiency of different social dentistry units was significantly different (12).

The aim of present study was to use a DEA model to assess, to rank and to identify the efficiency of dental units of Iran provinces based on some of the most important dental health inputs and outputs and finally to provide recommendations to improve Iran dental health situation.

## Methods

This was a cross-sectional, descriptive-analytical and applied study. Statistical population of this study included dental health sector (public and private) of all of Iran provinces. Because of the limited number of provinces, we did not use sampling.

After consulting experts and reviewing studies about assessment of different healthcare departments, three indices of Decay, Missing and Filled Teeth (DMFT) of 6, 12 and 15 yr old school pupils were selected as output variables and four variables of active chairs of public sector present in different provinces, general dentists of public sector, and general and specialist dentists of private sector of different provinces were selected as input variables (Table 1). The input data obtained from different universities of medical sciences across the country and the output data collected from 2013 national screening program for school pupils (13).

**Table 1:** Input and output variables needed to measure efficiency of dental units of Iran provinces using DEA method

**Inputs**	**Outputs**
Active chairs of public sector	Percent of decayed teeth
General dentists of public sector	Number of missing teeth
General dentists of private sector	Number of filled teeth
Specialist dentists of private sector	

Since the study, population does not work at optimal scale and by 1-unit increase in the inputs does not produce 1-unit more output, variable return to scale method used to assess efficiency. In this study, we used input-oriented model, because outputs were not in control of managers and they could only minimize the inputs to have more efficiency. In other words, managers only can manipulate the inputs to produce more outputs. However, as a general rule, the studied units or DMUs (here Iran provinces) should be at least 3 times more than the examined variables (inputs plus outputs), otherwise most of DMUs wrongly become efficient (14). In the current study, this rule has been respected; the numbers of studied provinces are 31 that is more than 3 times the number of the variables (which is 7). Thus, considering these three hypotheses (input-oriented, variable returns to scale and the number of DMUs), the linear programming problem to be solved is presented below. In this problem, K=4 (i.e. the study inputs), m=3 (i.e. the study outputs) and n=31 (i.e. the study DMUs). In addition, X is (k×n) input matrix and Y as the (m×n) output matrix. It is necessary to solve one problem for each DMU.

In this problem, *θ* range between 1 and ∞, and its inverse range between 0 and 1 which is the technical efficiency score.

$$\{\begin{array}{c} \min\theta (\theta ,\lambda )\\ -\mathrm{yi}+Y\lambda \geq 0\\ \theta \mathrm{xi}-X\lambda \geq 0\\ N1\lambda \leq 1\\ \lambda \geq 0 \end{array}$$

If it is equal to 1, the DMU is efficient, while if it is less than 1, the DMU is inefficient. λ is (n×1) vector of constants that measures the weights used to compute the location of an inefficient DMU if it was to become efficient. The model specification under the hypothesis of variable return to scale implies the condition of convexity of the frontier. This presumes that the restriction N_1_λ<=1 is introduced in the model, N_1_ being an n-dimensional vector of ones. The absence of this restriction implies that returns to scale were constant. In this study, we applied DEA model considering both the constant and variable return to scale and we also computed the scale efficiency for the DMUs in the sample. This is the ratio between the efficiency scores in constant and variable return to scale hypothesis and accounts for the increasing, decreasing or constant return to scale. The collected data were entered into Excel software and were analyzed by Deap software ver. 2.1.

## Results

The relative efficiency of different provinces in terms of dental health is presented in Table 2. Accordingly, provinces of Chaharmahal-and-Bakhtiari, South Khorasan, Ardabil, Ilam, North Khorasan, Kohkiluyeh-and-Boyer-Ahmad, Semnan, and Qom have both scale efficiency and managerial efficiency. While, provinces of Qazvin, South Khorasan, Ardabil, Ilam, North Khorasan, Kohgiluyeh-and Boyer-Ahmad, Semnan, and Qom have technical efficiency.

**Table 2:** Determination of scale, managerial and technical efficiency of dental units of Iran provinces using DEA method

**Province**	**S.E**^**1**^	**M.E**^**2**^	**T.E**^**3**^	**Province**	**S.E**^**1**^	**M.E**^**2**^	**T.E**^**3**^
Isfahan	0.205	1.000	0.205	Tehran	0.204	1.000	0.204
Razavi Khorasan	0.789	0.187	0.147	Chaharmahal	1.000	1.000	0.526
Gilan	0.952	0.232	0.220	Qazvin	0.894	0.588	1.000
East Azerbaijan	1.000	0.449	0.449	Sistan Baluchestan	0.413	0.721	0.298
West Azerbaijan	0.382	0.277	0.106	Bushehr	0.427	0.874	0.373
Kerman	0.402	1.000	0.402	South Khorasan	1.000	1.000	1.000
Mazandaran	0.993	0.299	0.297	Ardabil	1.000	1.000	1.000
Fars	0.532	0.186	0.099	Ilam	1.000	1.000	1.000
Zanjan	0.982	0.972	0.954	North Khorasan	1.000	1.000	1.000
Kermanshah	0.814	0.500	0.407	Kohgiluyeh	1.000	1.000	1.000
Hamadan	0.960	0.486	0.467	Alborz	0.492	0.545	0.268
Kordestan	0.952	0.698	0.664	Hormozgan	0.659	0.984	0.649
Markazi	0.991	0.781	0.774	Khuzestan	0.671	0.615	0.413
Golestan	0.876	0.587	0.514	Semnan	1.000	1.000	1.000
Yazd	0.572	1.000	0.572	Qom	1.000	1.000	1.000
Lorestan	0.671	1.000	0.671				

1 Scale efficiency

2 Management efficiency

3 Technical efficiency

Thus, although Chaharmahal-and-Bakhtiari has both scale and managerial efficiency, but it is not technically efficient. Although Qazvin Province has technical efficiency but has no scale and managerial efficiency. The lowest amount of scale efficiency was for Tehran Province (0.204) followed by Isfahan Province (0.205). The lowest managerial efficiency rate belonged to Fars and Razavi Khorasan, respectively. The lowest technical efficiency rate belonged to Fars, West Azerbaijan, and Razavi Khorasan, respectively.

Dental health sector of East Azerbaijan, Chaharmahal-and-Bakhtiari, South Khorasan, Ardabil, Ilam, North Khorasan and Kohgiluyeh and Boyer-Ahmad had constant return to scale. Provinces of Isfahan, Razavi Khorasan, Kerman, Zanjan, Hamedan, Kordestan, Golestan, Yazd, and Tehran had decreasing return to scale and provinces of Gilan, West Azerbaijan, Mazandaran, Fars, Kermanshah, Markazi, Lorestan, Qazvin, Sistan-and-Baluchestan, Bushehr, Alborz, Hormozgan and Khuzestan had increasing return to scale.

Table 3 indicates peer or reference provinces and their coefficients for inefficient provinces to reach the border of relative efficiency. For example, the peer provinces for Razavi Khorasan are Khuzestan, Bushehr and South Khorasan, so that their coefficients are 0.451, 0.388 and 0.161, respectively. The efficient provinces that their coefficient is 1, their peer provinces are themselves.

**Table 3:** Determination of peer provinces and their coefficients based on input-oriented method for dental units of inefficient provinces

**Province**	**Peer province 1**	**Coefficient of Peer province 1**	**Peer province 2**	**Coefficient of Peer province 2**	**Peer province 3**	**Coefficient of Peer province 3**
Isfahan	1	1.000				
Razavi Khorasan	31	0.451	23	0.388	24	0.161
Gilan	24	0.384	22	0.035	31	0.581
East Azerbaijan	23	1.000				
West Azerbaijan	31	0.333	26	0.197	25	0.470
Kerman	6	1.000				
Mazandaran	31	0.054	23	0.946		
Fars	31	0.000				
Zanjan	23	0.944	26	0.056		
Kermanshah	26	0.884	18	0.116		
Hamadan	31	0.376	23	0.624		
Kordestan	23	0.395	26	0.605		
Markazi	26	0.438	23	0.361	30	0.201
Golestan	31	0.230	23	0.770		
Yazd	15	1.000				
Lorestan	18	0.634	26	0.366		
Tehran	17	1.000				
Chaharmahal	18	1.000				
Qazvin	31	0.569	22	0.201	23	0.230
SistanBaluchestan	31	0.041	30	0.959		
Bushehr	22	0.339	26	0.626	25	0.035
South Khorasan	22	1.000				
Ardabil	23	1.000				
Ilam	24	1.000				
North Khorasan	25	1.000				
Kohgiluyeh	26	1.000				
Alborz	31	1.000				
Hormozgan	31	0.361	30	0.149	26	0.491
Khuzestan	31	1.000				
Semnan	30	1.000				
Qom	31	1.000				

## Discussion

Considering the fact that no holistic comparison has been performed between dental units of different provinces in terms of the efficiency of inputs to produce the best outputs with the lowest costs, doing this study seemed essential. In this study, the efficiency assessment performed using the most important inputs and outputs, so it is clear for policy makers to invest in which inputs in obtaining more outputs.

Applying DEA model by providing the suitable situation for comparison, ranking and modeling can create an important step toward continuous improvement of the country dental health sector. Using DEA in addition to determination of relative efficiency rate and organization weaknesses, by providing the desired level of performance indicators, can specify organization policy toward efficiency and productivity (15).

In this study, Provinces of Isfahan, Razavi Khorasan, Kerman, Zanjan, Hamedan, Kordestan, Golestan, Yazd, and Tehran had a better situation than other provinces in terms of the number of dentistry chairs, public dentists, general and specialist dentists of private sector, but they had decreasing return to scale. The mentioned provinces do not have a good situation in the field of technical efficiency (Table 2).

The optimal inputs should determine in order to shift inefficient provinces to efficiency boundary (Table 3).

In other words, as mentioned in the definition of efficiency, one way to improve efficiency is to decrease the inputs (number of active chairs, number of private general dentists, number of public general dentists and number of private specialist dentists) (Table 4).

**Table 4:** Determination of target inputs for inefficient inputs of dental units of different provinces based on input-oriented method

**Province**	**Number of active chairs**	**Target number ofactive units**	**Number ofprivate general dentists**	**Target number of private general detists**	**Number of publicgeneral dentists**	**Target numberof public general dentists**	**Number of prvate specialist dentists**	**Target number of private specialist dentists**
Isfahan	219	219.0	1706	1706.0	202	202	127	127.0
RazaviKhorasan	205	38.239	1490	133.404	137	25.555	135	3.966
Gilan	143	33.111	593	137.306	113	23.224	30	4.744
EastAzerbaijan	139	52.0	797	114.0	78	35.0	85	2.0
West Azerbaijan	137	38.001	429	118.996	98	21.713	13	3.606
Kerman	205	205.0	510	510.0	87	87.0	38	38.0
Mazandaran	169	50.495	828	116.312	119	33.817	49	2.215
Fars	129	24.0	1284	157.0	115	13.0	76	6.0
Zanjan	110	51.605	126	114.960	60	34.774	2	1.944
Kermanshah	99	47.089	272	129.375	65	30.768	2	1.0
Hamadan	99	41.461	280	130.185	55	26.719	24	3.506
Kordestan	94	47.768	202	124.277	63	32.582	2	1.395
Markazi	85	50.139	188	121.847	40	31.240	2	1.562
Golestan	80	45.551	300	123.904	51	29.933	9	2.291
Yazd	80	80.0	329	329.0	55	55.0	8	8.0
Lorestan	74	56.414	188	122.122	39	29.732	1	1.0
Tehran	202	202.0	9665	9665.0	213	213.0	1036	1036.0
Chaharmahal	63	63.0	117	117.0	29	29.0	1	1.0
Qazvin	62	36.467	219	128.811	47	21.764	9	4.478
Sistan&Baluchestan	86	56.616	209	117.669	34	24.512	3	2.163
Bushehr	55	48.047	123	107.451	67	30.755	2	1.747
SouthKhorasan	54	54.0	66	66.0	31	31.0	3	3.0
Ardabil	52	52.0	114	114.0	35	35.0	2	2.0
Ilam	45	45.0	114	114.0	38	38.0	3	3.0
NorthKhorasan	45	45.0	87	87.0	24	24.0	3	3.0
Kohgiluyeh	45	45.0	131	131.0	31	31.0	1	1.0
Alborz	44	24.0	1198	157.0	45	13.0	123	6.0
Hormozgan	40	39.360	143	138.146	24	23.616	3	2.952
Khuzestan	39	24.0	589	157.0	39	13.0	30	6.0
Semnan	58	58.0	116	116.0	25	25.0	2	2.0
Qom	24	24.0	157	157.0	13	13.0	6	6.0

Policymakers should consider that simply development of physical and human resources cannot improve DMFT and other dental health indices. Only providing resources are not adequate to ensure improvement. For example, lack of insurance, low family income, low parents health literacy was identified as main causes of lack of dental examination (16).

The mentioned factors are necessary for access to dental healthcare (17). Some of less costly strategies for dental health promotion are establishment of NGOs to address dental health demands of the community, knowledge promotion and community education (18).

Purchasing expensive dental equipment and establishment of dental schools are not in line with the priorities of WHO. In addition, training of dentistry students in Iran had not been targeted toward the real needs of society. WHO has presented essential package of dental care that is to be integrated into the local health care services, the dental needs of the population be met (19). Restoration of permanent teeth of children in the low-income countries using dental amalgam cost between 1618–3513 USD per 1000 children of mixed age group of 6–18 yr old. This amount is far greater than available resources to provide an essential package of health services for 15–29 yr old age group in the low-income countries (20).

Government planning to improve dental health literacy is much more effective and less costly than investing in equipment and specialized fields. In addition, whatsoever oral health literacy is lower, dental disease is more severe (21–23). In a study on determinants of oral health in Iran, low oral health literacy level is a predictor of poor self-reported oral health and should be considered a vital determinant of oral health in countries with developing health care systems (24). During the last decade, without considering the necessary infrastructure and providing adequate faculty members, the number of dentistry schools has increased and preventive dental health care has been neglected. On the basis of 2013 statistics, 37 public medical universities in Iran have admitted 880 dentistry students, international campus of 18 medical universities have admitted 270 dentistry students and 5 Islamic Azad Universities (private schools) have admitted 235 dentistry students. In 2013, 1385 dentistry students have been admitted to Iran universities, totally (25). The cited statistics have not included the data of Iranian dentistry students who are studying abroad. Certainly, the vast majority of these students will return to the country after graduation.

Based on 2008 European Union data, European countries had the average of 1 dentist per 1408 people (26). In 2012, this figure was 1 dentist per 3000 people in Iran (27). However, with the rapid growth of Iran’s dentistry students, if there is no comprehensive plan to deal with this phenomenon, Iran will get the first rank in dentist to population ratio in the near future. A large number of dentists in the country is only one side of the case. Maybe the more important problem is their distribution all over the country that might strengthen inequities in this area. As satisfaction and retention of health professionals in less developed regions have been mentioned as a challenge in previous studies (28, 29). The density of dentists in Iran is in better-off provinces. In other words, people with better social rank have more access to dentistry services (30).

## Conclusion

In spite of investments made to improve oral health, but they have not been efficient. Iranian health system has ignored less expensive and cost effective first level interventions and has mostly focused on providing inputs for second and third level services. The present trend of training dentists is constantly increasing dentist to population ratio that in turn might deviate scarce resources provided for oral health to expensive interventions. Therefore, it is necessary for policymakers to take some measures to improve efficiency in using oral health resources.

The data on dental units were collected from medical universities, which are officially responsible for supervision of dental services delivery. Since a number of unsupervised dental chairs exist in the country, especially in Tehran province, the results should be interpreted cautiously.

## Ethical considerations

All the authors carefully observed ethical consideration regarding performing and disseminating the results including the ethical issues of taking the data, avoiding plagiarism, authorship, etc.
